# Quality of care in abortion in the era of technological and medical advancements and self-care

**DOI:** 10.1186/s12978-022-01499-3

**Published:** 2022-09-15

**Authors:** Ankita Shukla, Lucía Vazquez-Quesada, Isabel Vieitez, Rajib Acharya, Saumya RamaRao

**Affiliations:** 1 University of Sharjah, Sharjah, UAE; 2 Population Council, Mexico City, Mexico; 3 Population Council, New Delhi, India; 4 Population Council, New York, USA

**Keywords:** Abortion, Quality of care, Telemedicine, Medical abortion, Pharmacists, Misoprostol, Abortion self-management

## Abstract

**Background:**

Discussions around quality of abortion care have been focused mainly on service-delivery aspects inside healthcare facilities. More recently, with availability of medical abortion (MA), increase in its self-use, and emergence of other delivery platforms such as telemedicine, the responsibility of quality care has broadened to actors outside of facilities.

**Body of text:**

This commentary discusses the meaning of quality of abortion care with the paradigm shift brought by medical and technological advancement in abortions, and raises questions on the role of the state in ensuring quality in abortion management—especially in settings where abortion is decriminalized, but also in countries where abortion is permitted under certain circumstances. It consolidates the experience gained thus far in the provision of safe abortion services and also serves as a forward-thinking tool to keep pace with the uptake of newer health technologies (e.g., availability of medical abortion drugs), service delivery platforms (e.g., telemedicine, online pharmacies), and abortion care providers (e.g., community based pharmacists).

**Conclusions:**

This commentary provides context and rationale, and identifies areas for action that different stakeholders, including health advocates, policymakers, program managers, and women themselves, can adopt to fit into an alternative regime of abortion care.

**Supplementary Information:**

The online version contains supplementary material available at 10.1186/s12978-022-01499-3.

## Background

Technological and medical advances in the provision of abortion care have shifted the classic dynamic between providers and users, with implications for the service-giving process and quality of care. Today, pregnant individuals can safely seek an early abortion remotely using telemedicine or websites like Women on Web (www.womenonweb.org), or relying on trained peers for information and support [1]. The self-administration of medication abortion broadens the possibilities in terms of who manages the abortion, when, and by what means, especially in liberal settings, but also in legally restrictive contexts. In 2021, approximately 1.6 billion women worldwide were living in a country where abortion is allowed under at least one legal indication, 90 million (5%), live in a country where abortion is prohibited altogether [2]. But this prohibition does not prevent women^1^ from seeking a pregnancy termination and there are reports suggesting that even in settings of total prohibition, availability of medication abortion drugs via pharmacies seems to be associated with a reduction of abortion-related morbidity [3]. Trends in settings that are restrictive, but do not outright ban abortion, also suggest that women are increasingly replacing risky methods of abortion with medical abortion drugs [4]. Pharmacists and nonphysical cadres are emerging as the first points of information and care for medical abortion because of their accessibility to communities and the privacy they offer to women seeking abortions across settings [5].

So far, discussions on abortion have included structural, institutional, and individual factors in the provision of abortion and post-abortion services [1, 6, 7], but the discussion on quality of care is limited to in-service delivery and/or the quality of technical safety and efficacy of the abortion procedure [8]. Questions on the quality of abortion provision and management outside of health facilities has been overlooked, and has not explicitly included a rights and health-system perspective, i.e., an approach inclusive of all health stakeholders ranging from health systems and organizations to individuals working to promote, restore, or maintain health.

In the literature, the client’s experience and satisfaction is often used as a proxy measure of quality. However, women’s satisfaction can be an adequate measurement of quality only if women are aware of their rights and available options, in the absence of which their perception of quality is biased [8]. We thus argue that quality of abortion care needs a broader framework inclusive of rights and health-system perspectives along with the advancements in abortion services such as self-care. The Abortion Service Quality Initiative group (https://asq-initiative.org/) is working on a similar line to develop a common measure of abortion service quality in order to assess service provision within facilities as well as at out-of-facility locations.

In this commentary we discuss questions such as: What does quality of care mean with the paradigm shift in the technological advancement in abortions? What should the role of the state be in ensuring quality of abortion (that is safe, effective, user-centered, timely, efficient, and equitable) in settings where abortion is legal or allowed under certain legal indications—and thus more restricted access?

### Quality of care in abortion (A-QoC)

In an era where abortion drugs are accessible and self-managed abortions are possible, a comprehensive systems approach is needed to assess quality of abortion care. A systems approach considers “all organizations, people and actions whose primary intent is to promote, restore or maintain the health” [9], including all steps necessary for making information, products, and care available to people (regardless of whether they seek an abortion or not). A state’s governance should ensure that abortion care providers inside and outside of healthcare facilities have the necessary information and training to communicate about the procedure in confidential, respectful, and non-stigmatizing ways, and to regulate manufacturing and availability of quality drugs. We argue that to achieve quality abortion care, a rights-based approach is necessary to safeguard individuals’ entitlements to quality care, dignity, and respect, acknowledging the diversity and intersectionality of individual experiences in the access to and use of abortion services. Holistic quality care calls for a system as well as relational and individual-level care. Pregnant individuals currently seeking abortion, or those who may have a future need, should be at the center of focus to indicate that their needs and voices are paramount in the design, deployment, and delivery of services. If abortion is destigmatized and information is readily available, individuals who have a future need will know where to go and whom to ask. A way of incorporating those who might need an abortion service might be consulting the needs of women and other pregnancy-able individuals receiving family planning services, or of individuals who accompany a woman in their search for abortion services.

Based on the available literature and our own research related to quality and safe abortion care, we lay out here important domains that need to be in focus in discussions of quality of care in abortion. A detailed list of components under each domain is given in Additional file 1: Box 1.

### Enabling political, legal, and socio-cultural environment

Legal frameworks are an important factor for the availability and accessibility of information and abortion services. The more liberal the legal framework and the less stigmatizing the socio-cultural environment, the better the quality of abortion care. Evidence indicates that legal restrictions on abortion do not prevent its incidence, but rather make it unsafe [10]. Liberal abortion legislation is associated with increased information, access, and quality of self-induced abortion services with lower levels of abortion stigma [11, 12]. Evidence from several settings encourages removal of legal, policy, and bureaucratic barriers, and an increase in awareness of rights to safe abortion services [10, 11]. However, the process of reformation in abortion law is complex and may take a long time [11].

From a human-rights perspective, quality standards of care need not vary across countries. Criminalization and stigmatization affect State’s stewardship and directly affect equity in the provision of quality of care. Early medical abortion services are provided by various actors, and depending on the context, they do so openly or surreptitiously. Our point is that these actors are not exempt from considering quality in their provision of care, and so our hope is that this commentary helps them to think about the dimension needed to provide the service (or their part of the service, i.e., selling the medication) with increased quality. In many settings with restrictive abortion rules, enhancing quality of care is framed as a harm-reduction strategy. Likewise, even when self-managed abortion is not regulated, the provision of information is justified under a harm-reduction strategy, as is the availability of post-abortion care. The World Health Organization (WHO) has recommended providing adequate information about misoprostol even in restrictive settings, since people will use the method with or without correct information [13].

Countries where abortion is legal for at least one indication should ensure that information on medical abortion is available to pregnant individuals. Provision of correct information on effective use of MA or referral to authentic sources of information can be provided via healthcare professionals, local NGOs, or women’s organizations, among others, recognizing that these actors are upholding women’s rights to information. The argument can hold for settings where abortion is completely banned, depending on whether countries have signed international treaties that safeguard sexual and reproductive rights. Provision of information and not the service itself would not be violating any laws.

In many regions of the world, regardless of the legality of abortion, stigma prevails and determines how the services are provided. Abortion stigma acts overtly or subtly as a barrier to ensuring timely access to—and quality of—comprehensive and people-centered abortion care, as women may not seek or access care at all or may delay it due to fear of social punishment [14, 15].

A socio-cultural environment that enables quality in abortion care works at the macro (media, culture, and legal framework), meso (institutions and communities), and micro (individual) levels to reduce abortion stigma [16], and prioritizes the dissemination of updated scientific information on the use of abortion technologies and processes of care (including self-care) for all legal indications for abortion. In addition, it ensures evidence-based policies and programs to provide the service in clinical settings, which can further ensure equitable access to quality abortion care [11].

### Structure/health system/role of state

The provision of good-quality care is predicated on the existence of a functional health system that sets standards and guidelines for healthcare providers and provides them with the necessary knowledge, equipment, resources, and infrastructure for service provision both inside and outside of healthcare facilities. States can shape the health system through their role in government and ministries of health, as policymakers, and with technical assistance to actors within the health system—including health promoters, pharmacists and chemists, and local organizations. Government and ministries of health can influence the framing of evidence-based policies.

In democracies, even in weak ones, the state is not monolithic, and even when stigmatization and criminalization exist, there is opportunity for state institutions to support quality of care via harm-reduction strategies. Before abortion decriminalization, for instance, Uruguay applied a harm-reduction strategy consisting of giving pregnant women with a high-risk pregnancy public domain information allowing them to decide about the pregnancy, and if they chose abortion, to proceed to conduct a lower-risk procedure using misoprostol. This strategy started off in a hospital and scaled up nationally [17]. Countries like Nigeria and Rwanda, where abortion is restricted, have found room to include MA drugs on their essential medicine list. Recent developments in Mexico are a good example of how governmental institutions are able to exercise their power to allow for abortion even when it is restricted by law. In 2021, the nation’s Supreme Court of Justice declared that criminalizing women for inducing an early abortion affects their rights, so even though abortion on demand is not legal in this federal country—and thus not provided in health-facilities across Mexico—the Supreme Court provided legal arguments to prevent those women and other individuals able to get pregnant who induced an abortion from going to jail. These examples show that among state institutions and providers, there is scope for progressive change, but they need help and support from advocates and the public health community to identify which door to open. The accompaniment models on abortion in Latin America are also good examples of this [18]. Accompaniment models on abortion vary but they basically consist of trained grassroot organizations or activists providing pregnant individuals with evidence-based counseling and support through the medication abortion process outside of clinical settings.

### Leadership governance

WHO has developed evidence-based policy strategies and technical and nontechnical guidelines to promote abortion and post-abortion care, which provide the framework that national health systems can follow. WHO has designated governance as “stewardship,” whereby oversight, regulation, incentives, and accountability are based on the principles of equity and transparency [19]. At the national level, a government’s stewardship and oversight role on abortion care should ensure the existence of a strategic health policy framework on sexual and reproductive health (SRH) [20], including abortion. Policies can (and must) focus on:

1. *The prevention of unsafe abortion and complications*
2. *Ensuring equal access to legal and safe abortion*
3. *Oversight of services to ensure safe and effective, good quality, and timely services across sectors (public, private, remote, or provided by a health or non-health professionals, including telemedicine)*
4. *Strengthening the supply chain and logistics to ensure that abortion drugs and associated supplies are available at the times individuals need them and at accessible points of services*
5. *Establishing indicators to monitor and evaluate progress*

#### Sustainable supply chain

Delay in the provision of key medicines and antibiotics or stockouts of medical abortion drugs increase risks to women’s health [21]. Strengthening the supply chain and logistics will ensure that quality drugs, equipment and associated supplies needed for abortion are available at the times individuals access services. This is central to the provision of quality abortion care both inside and outside of healthcare facilities.

Arranging adequate funding and capacity for supply management, will ensure that the gain achieved by the introduction of technological and medical advancements are not reversed, as happened in Malawi and Cameroon where, due to the unavailability of manual vacuum aspiration kits after a pilot intervention, use of dilation and curettage resurfaced [22]. Procurement and distribution systems should be strengthened to avoid stockouts of medical abortion drugs that have been an obstacle to expanding medical abortion services in several countries. State stewardship is essential for a supportive policy environment with adequate financial investment to strengthen the supply chain system to ensure the availability of medicines and to avoid stockouts and the expiration of unused products. National governments should work to promote public-sector availability and competitive pricing in the private marketplace, and to subsidize access to medical products for poorer and marginalized populations.

The Reproductive Health Supplies Coalition (RHSC) highlights four key strategies to enhance supply chain and logistic functions: (1) integration of supply chain into a single logistics system for all essential medicines; (2) strengthening the public–private partnership to address bottlenecks in the supply chain and to improve access to high-quality supplies; (3) establishment of security committees to bring together multiple stakeholders to support enhanced coordination, address long-term product availability issues, and reduce duplication and inefficiencies; and (4) development of supply chain tools such as databases and information platforms in order to have comprehensive and timely information.

#### Financing

Even as countries undertake health-sector reforms and implement universal health coverage (UHC), safe abortion services are typically not included [23]. In contexts where health financing is private and voluntary, out-of-pocket payments can exacerbate existing gender differentials in access to and use of health services [24]. Healthcare systems in many low- and middle-income countries are not well financed to provide the full range of quality SRH services. Often such settings maintain a list of priority healthcare services that are made available at no or subsidized cost. This priority list rarely includes services related to safe abortion or comprehensive SRH awareness, education, and/or counseling [25]. Lack of resources combined with a paucity of political commitment are major obstacles in providing universal access to SRH services in such settings [26].

Even in contexts where health financing is public and compulsory and great progress has been made to ensure health services (such as in Thailand), access to safe abortion services remains a challenge [24]. All of these suggest the importance not only of ensuring adequate financing but also of paying attention to the design and implementation of the financing approach to ensure essential rights to health [23, 27].

In settings with liberal abortion laws self-management of abortion should be a part of an active policy to increase universal health care coverage and reduce inequalities. On the other hand, in restrictive settings health financing should include harm-reduction strategies such as information on self-care for abortion and the availability of quality MA drugs [28].

Reducing the costs of services both inside and outside of health facilities, such as expanding access to quality generic MA formulations, while facilitating access to all should be an integral part of health system preparedness to provide quality abortion and post-abortion care. National health systems should identify and prioritize cost-effective and innovative interventions such as performance-based financing and community-based health insurance [29], government subsidies, private financing, and insurance coverage catered to specific populations.

#### Mechanisms for accountability, transparency, and monitoring

Data on abortion are limited and incomplete in most settings, and in locations where abortion is restricted it is even harder to obtain data due to the large number of clandestine abortions. This makes it difficult to ascertain the magnitude of the issue, identify service gaps, and monitor the improvements implemented to improve care. Compounding the problem of incomplete data is the fact that there is no consensus or common definition of quality of abortion care despite a plethora of indicators suggested by researchers to measure different aspects at the structure, process, output, and outcome levels [30, 31]. A well-functioning health system needs to monitor and evaluate the quality of abortion and post-abortion services inside and outside of healthcare facilities. The indicators used to monitor progress should be meaningful and useful to all stakeholders—individuals seeking or self-managing an abortion, medical staff, health ministries, legislative bodies, local governments, and private sector, civil society, and nongovernmental organizations—to ensure accountability.

The Global Abortion Policy Database is an example of mechanisms that encourage transparency and state accountability by providing a worldwide and comparative repository on national laws, policies, health (and human rights) standards and guidelines [32]. Similarly IPPF’s Medical Abortion Commodities Database provides country-level information on the availability of different brands of mifepristone, misoprostol, and combi-packs and their quality (https://www.medab.org/about-this-database) that can be used by different stakeholders working on abortion.

### Service delivery process both inside and outside of health facility (quality offered)

From a health systems approach, the quality of abortion services is fostered and supervised at each delivery point, such as clinics, pharmacies, homes, teleservices, and/or the internet. The service delivery process should encompass all remote and face-to-face interactions between abortion-seekers and providers (healthcare providers, counselors, pharmacists) who provide information or the service or who sell the medication abortion drugs. In the following, we describe the six essential elements that are required to ensure that good quality care is offered to pregnant individuals seeking an abortion.

#### Availability of abortion type and providers

Providing choices to consumers is an integral part of any health-service delivery inside or outside of healthcare facilities. Having been provided with choices, individuals can select the method that best fits their needs, preferences, and circumstances, including the time and financial resources available to them. Studies have reported that women choose one method over another depending upon their personal preference formed by perceived risk, emotional impact, need for privacy and control, physical ability to bear pain, and their confidence and trust in their own bodies [33, 34]. Choice of service-delivery points depends upon information about different care options, waiting time, distance to access point (e.g., clinic, pharmacy), work or childcare commitments, eligibility for free services, privacy, and prior experiences of abortion care or other health care [35, 36]. Hence individuals should be given the choice of accessing abortion services both inside and outside of health facilities. Health systems which do not provide options limit individuals’ likelihood of opting for a safe method per their need. Clearly restrictions do not always prevent people from seeking abortion, thus even in places with restrictive abortion laws women should at least be aware of choices and provided with information on self-management of abortion so that they will not resort to unsafe options.

#### Information exchanged

To ensure that women have the necessary information to make an informed decision, providers inside and outside of health facilities should counsel women seeking care about the options available to them, details of the procedures, the pros and cons of each procedure, related complications, costs, and any other information available according to each woman’s unique needs and circumstances. Information exchange between the provider and the client should be a two-way communication process. Service providers are expected to create an environment where clients feel respected, enable them to share their doubts, and encourage them to ask questions. Women seeking abortion have reported that when providers have discussions with them and ease their anxiety, it makes their experience much better and less frightening [37, 38]. Pre-abortion counseling is also beneficial for individuals who have already made their choice, as many times their decision is based on incomplete or erroneous information gathered from peers or other nonprofessional sources and their prior abortion experiences [33, 39, 40]. However, even professionals such as pharmacists have rarely been observed to provide in-depth details while selling MA drugs and there is rarely a provision for sensitizing pharmacists on these issues [41, 42]. Abortion service seekers must be told what to expect during the process, what warning signs indicate possible complications and what emergency care is available, and about post-abortion family planning.

As the number of service outlets—both online and physical—increases, there is a risk that providers on these platforms will not have in-depth conversations with abortion seekers. Policymakers and abortion advocates must take into consideration these additional sources for abortion information and services, in culturally appropriate and localized languages, to ensure the timely provision of updated, evidence-based, and nonjudgmental information on abortion for pregnant individuals. From our viewpoint, websites and other dissemination channels would provide a better service when including ways to create a two-way communication process (e.g., a telephone, chat, of FAQ that is constantly revised and updated to address users’ needs). Means to conduct confidential and remote pre-abortion and post-abortion counseling should also be made available to the individuals. To ensure that individuals have the necessary information to make an informed decision, providers inside and outside of health facilities should counsel individuals seeking care about the options available to them, details of the procedures, the pros and cons of each procedure, related complications, costs, and any other information regarding their unique needs and circumstances.

#### Interpersonal relationship

Abortion-care providers should uphold the values of nonjudgment, privacy, confidentiality (characterized by demonstrating trust), respect, empathy, cordiality, and clarity. The interpersonal communication between the trained provider and the pregnant individual should take place in a private and comfortable space, and providers should use nontechnical, easy-to-understand language. Effective communication between provider and client helps optimize the abortion process. In-service mistreatments of individuals seeking an abortion include perceived or actual verbal harassment, deliberate delays, or denial of pain medications [43].

#### Technical competence

Technical aspects of abortion care, including healthcare providers’ skills and well-equipped facility are deemed to be important elements in measuring quality of abortion services in clinical settings [44]. Knowledge gaps among healthcare providers have been reported regarding new methods like MA drugs [45], in tackling emergency cases, and on referral services [46, 47]. Service providers should be competent in pregnancy determination, pre/post-abortion counseling, gestation assessment, medication dispensing, procedure performance, assessment of completion of abortion, patient monitoring, and follow-up assessment and care [48, 49]. Often, healthcare providers’ training is focused on the clinical aspects of the caregiving with less attention to process and psychological elements. Furthermore, despite being trained, providers’ skills can diminish with low caseloads, hence refresher training is recommended. In addition, healthcare facilities should be well equipped with all the necessary abortion care–related equipment, acceptable infection prevention mechanisms, and waste management to support providers in the delivery of care.

Pharmacy workers and drug sellers have been documented to have poor knowledge of effective regimes of medication abortion, side effects, and complications resulting from MA drugs, and are thus prone to recommending under/over-dosage leading women to have incomplete abortions or harmful post-abortion complications. Additionally, evidence suggests that such providers either have no knowledge of the national guidelines on medication abortion or choose not to follow them [50].

Non–facility-based providers such as pharmacists, community drug store operators, telemedicine providers, and women’s organizations that dispense medication abortion pills should be mandated to offer women basic knowledge about the abortion process and related potential complications. Such providers should know—and clearly explain—how to determine pregnancy and contraindications and the correct combinations and doses of the drugs, and provide information on referral points and contact numbers in case of emergency. Training in the clinical and social aspects of abortion care is therefore needed for all abortion service providers and appropriate step-by-step referral mechanisms should be in place to help both in-facility and out-facility providers in the event of complications.

#### Follow-up mechanism

After an abortion, women should receive clear oral and written instructions for post-abortion care to help them understand what happens once the procedure has been completed. These include post-intervention hygiene, confirming the completion of a medication abortion especially if it occurs at home, how to identify complications, if any, return of fertility and resumption of sexual relations, protection from STIs, post-abortion contraception (PAC) counseling, and provision of contraceptive methods [51, 52]. The frequency, duration, and continuity of the optional follow-up visits should be designed according to women’s needs and preferences [53, 54]. With increased mobile penetration in the communities, remote follow-up after medical abortion to confirm completion is a feasible, safe, and acceptable alternative.

Intervention research conducted in multiple settings has demonstrated that providing contraceptive counseling and services at the same time and location as post-abortion treatment can rapidly increase immediate PAC acceptance [55]. If contraceptive methods are not available on site (or for providers outside of medical facilities), there should be a direct referral system to ensure that women are able to obtain their chosen contraceptive method [20]. One of the barriers to PAC is that in many countries abortion services are delivered at the second level of care and contraception at the first level of care; most women do not follow-up with their primary healthcare provider. This highlights the need to include contraception services at the second level of care, too.

#### Constellation of services

*Family planning* services are an essential part of the constellation of services that accompany abortion care. Along with abortion care, both in-facility and out-of-facility providers should offer PAC counseling and services to women before they leave the service-delivery point even in the case of online services [20].

*Screening for STIs including HIV*, HPV, and cervical cytology can also be part of the in-facility services offered to pregnant individuals who have had an abortion, as it may take less time than targeted testing. Moreover, given that intimate partner violence is a risk factor for unintended pregnancy and abortion [56], and that such experiences are often underreported, it is important to take the opportunity of women coming for abortion care to screen, counsel, and refer them to various services such as shelter homes; hotlines; and legal, health, and social services. Care should be placed in preventing such screening from being stigmatizing [57]. Since it may not be possible to provide the entire constellation of services at the same point of care, effective and efficient referrals to accessible services should be made. Awareness, referrals, and information on outlets for the additional services are even more critical when women seek abortion services from pharmacists, online platforms, or through telemedicine.

### Individuals (users and non-users)/partners/peers

Individuals should always be at the center of programming decisions and the emphasis should be to meet their needs on their care-seeking journey—at the point of care they desire to seek information and services from, information that is comprehensible for autonomous decision-making, and mechanisms that support their rights to health including creating awareness of available safe abortion services and the possibility of self-managing an early abortion. Healthcare systems that offer person-centered care (PCC) have been linked with positive patient experiences and improved adherence to care and treatment [58].

To provide a stigma-free and quality abortion experience partner and/or peer support is paramount. Stigma and negative attitudes toward abortion within the community and family affect the decision-making of the individual seeking an abortion. With regard to abortion care, PCC calls for efforts to reduce the internalized stigma that individuals who are seeking or have sought an abortion might experience. Quality of care is reflected in targeted communication strategies that effectively increase knowledge and sensitization, and build awareness about abortion in the community, thereby reducing stigma and access barriers when women need abortion services.

#### Awareness of laws/rights/methods/service locations

The WHO guidelines on safe abortion consider correct knowledge among both women and providers of the legal status of abortion to be an indicator for measuring access to information about safe abortion [20]. Despite the self-evident nature of the importance of awareness, in many settings women’s level of knowledge of the legality of abortion is very low [40, 43, 59, 60]. Because of uncertainty about the law and a lack of information about available methods and service delivery points, women often resort to sources such as drug sellers, traditional healers, and untrained providers [43, 61, 62]. They may delay their care-seeking to the extent that they arrive at service points with abortion complications or at gestations beyond the legally permitted limits. This increases the likelihood they will access unsafe services outside the legal health system. Clearly there is an unmet need for public education on rights to termination of pregnancy. The lack of knowledge is one of the major barriers to timely access of abortion options that are safe and that meet individuals’ needs. Furthermore, we believe that self-administration of abortion drugs must be a preference—a choice—and not a last resort, especially in settings where abortion is less restrictive or decriminalized.

#### Equitable access to services

Unsafe abortions are often the consequence of social determinants and different health opportunities among individuals and groups, regardless of whether abortion is restricted or not [63]. This is supported by the fact that unsafe abortions are disproportionately high in low-income settings and among unmarried women. In some countries unmarried women are more likely than their married counterparts to seek services from informal providers, despite being aware of the dangers of unsafe abortion, to avoid the stigma and judgmental attitudes of society and providers [64, 65].

The clustering of unsafe abortion practices among certain disadvantaged groups and the failure of the formal health system to ease their fears and concerns highlight the social injustice and health inequity in access to safe abortion services. Supportive state policies, insurance coverage and expansion in service delivery points, service subsidies, and integration of services into regular health care should be measures that health systems use to reach marginalized and disadvantaged individuals.

## Conclusion

As a result of new technical and medical advancement, their accessibility to communities, and the privacy they offer to clients (as well as their effectiveness and satisfactory performance), the nature of standard abortion services has changed. This opens up the opportunity to self-manage, task-shift and task-share abortion provision. In addition, technological developments make it possible to provide remote services through telemedicine and abortion websites/hotlines, all of which have the potential to alleviate pressure on health systems with limited human resources. In this context, understanding what quality of care entails is relevant for a series of stakeholders providing abortion services both inside and outside of health-care facilities. Thinking about quality of care in abortion requires expanding our view on the connections between actors in health systems and how they can synergize each other’s efforts. Whether providing one-way or two-way information, medical drugs by pharmacists/websites, or in-clinic services, all actors should ensure that the processes they are in charge of are safe, effective, user-centered, timely, efficient, and equitable.

The state must provide stewardship to facilitate this happening based on the most up-to-date scientific evidence throughout the dimensions identified in this commentary, even if conducted through a harm-reduction strategy in settings where the legal framework is restrictive for abortions on demand. The current policy and legal context of the state governs the provision of health services in any setting. However, it is established that irrespective of a state’s obligations, services are being provided, hence there is room for maintaining the quality of abortion services (e.g., WHO recommends use of misoprostol only if use of mifepristone is not approved). Provision of adequate information, especially for those opting for services outside the health system can be done via many nonconventional sources such as local NGOs, women’s organizations, websites focusing on women’s health, and sensitizing pharmacists and other community health workers. States could allow this to occur. Discussions about safety and efficacy in the use of medical and technological advancements in abortion care will influence the shift in the public perception of newly available methods.

Reducing maternal mortality is a priority for almost all low- and middle-income countries. States wanting to reduce maternal mortality but reluctant to address abortion, need to resolve their internal incoherence. Furthermore, the rollout of universal health coverage (UHC) provides the opportunity to integrate abortion services more meaningfully with relevant reproductive health programs. We anticipate that more individuals will adopt self-care behaviors and new health technologies to manage their health than ever before. The ongoing COVID-19 pandemic has emphasized the necessity and benefits of self-care strategies. Evidence from European countries reveals that demand for self-managed abortions has increased since the onset of the pandemic [66]. This commentary consolidates the learnings gained thus far in the provision of safe abortion services and advocates for forward thinking to keep pace with the greater availability of medical abortion drugs, new service delivery platforms (e.g., telemedicine, online pharmacies) and abortion care providers (e.g., community-based pharmacists). We hope that our discussion will provide context, rationale, and areas of action that stakeholders such as health advocates, policymakers, program managers, and individuals can engage and act upon to ensure good quality care to pregnant individuals—regardless of whether they seek a self-administered abortion, a remote consultation with a healthcare provider, or to visit an abortion clinic. When stakeholders act in concert, pregnant individuals wishing to terminate an unintended or unwanted pregnancy will be able to do so safely and effectively, and with dignity and respect. More importantly the rights of individuals to care will be promoted and protected.

## Supplementary Information

**Additional file 1.** Domains of quality of care in abortion (A-QoC).

## Data Availability

Not applicable.

## Abbreviations

HIV: Human immunodeficiency virus
HPV: Human papillomavirus
MA: Medical abortion
PAC: Post abortion contraception
PCC: Person-centered care
PAC: Post-abortion contraception
QoC: Quality of care
SRH: Sexual and reproductive health
STI: Sexually transmitted infection
UHC: Universal health coverage
WHO: World Health Organization

## Footnotes

1 Abortion care is needed for all individuals who seek abortion services whether for themselves or their partner/peer. Throughout this paper, however, we sometimes refer to “women” (instead of “pregnant individuals or those who may have a future need”), as they are the main group requiring abortion and post abortion-care, and most of the available research has been done on “women.”.
