# Supplementary material for: Quality of care in abortion in the era of technological and medical advancements and self-care
Source: Reprod Health. 2022 Sep 15;19:191. doi: 10.1186/s12978-022-01499-3 (PMC9479303; doi:10.1186/s12978-022-01499-3)
Supplement: Supplementary file 1 — Additional file 1. Domains of quality of care in abortion (A-QoC). [file 12978_2022_1499_MOESM1_ESM.docx]

***Box 1: Quality of Care in Abortion (A-QoC)***

**Enabling political, legal, and socio-cultural environment**

- - - - *Community awareness of reproductive rights/gender equality etc. (targeted communications strategies to increase knowledge and build awareness about gender equity and human rights)*
- *Cultural norms (norms that support equity, social and human rights, and inclusion)*
- *Legal framework (comprehensive and stigma-free legal frameworks that are accountable to human rights and gender perspective)*
- *Trusted and credible abortion care information [TRUSTED INFORMATION THROUGH DIFFERENT CHANNELS (WEB-BASED, PRESS, ETC BEYOND POINTS OF CARE]*

**Structure/Health system (Policy and program readiness)**

- *Leadership governance (State stewardship mechanisms for effective governance in both public and private sectors)*
- *Financing mechanisms (Institutionalized financing mechanisms)*
- *Sustainable and equitable supply (sustainable and cost-effective procurement and resupply mechanisms for essential drugs and supplies)*
- *Mechanisms for accountability, transparency, and monitoring*

**Service delivery process both inside and outside facility (Quality offered)**

- ***Availability of abortion type and providers***
  - - *Availability of full choice of uterine evacuation devices, methods, and medical products (MVA, EVA, MA)*
    - *Choice from individual’s perspective (type of method/ health effects/ ease of access/ privacy / providers’ attitudes/Cost)*
    - *Availability of providers*
- ***Information exchanged****- both provider and client need to exchange information*
- *Clients – on reproductive history, parity, age, other related information, or concerns*
- *Provider (both inside and outside facility)- encourages client to ask questions the choice client has, pros and cons of each method, the abortion process, prescription of drugs, about complications, costs.*
- ***Interpersonal relationship*** *(respectful interpersonal relationships & communication are practiced by all staff and persons in contact with pregnant individuals currently seeking abortion or those who may have a future need)*
- *Medical and non-medical providers’ sensitiveness towards issue of abortion (Non-judgmental attitudes, privacy and confidentiality, friendly behavior)*
- *Use of culturally appropriate and non-technical language and ensure that the client understands the information*
- ***Technical competence*** *(both inside and outside facility medical and non-medical providers are well trained to provide information & attention to user and adequate and timely referral -if needed)*
  - - *Availability of critical medicines and equipment, trained provider, functional OT, bed, testing facility, competent staff to tackle complications, referral mechanism, Infection prevention mechanism, waste management*
    - *Availability of FP counseling, FP methods, contact numbers in case of emergency*
    - *Technology update for related staff*
      - *Outside facility (Informal provider/Chemist) -Competency and knowledge of MA, Knowledge of referral points*
      - *Outside facility (Telemedicine)- Trained provider on call/chat, list of referral facilities available*
- ***Follow up mechanism*** *(both inside and outside facility providers provide all the information to women to ensure post-abortion care & access the constellation of services they need in the continuum of health care)*
  - - *Provide contacts (e.g.- phone numbers) and step by step information in case of emergency/ needing additional information*
    - *Options and instructions given to clients for virtual or in person follow-up visits (time, number)*
    - *Confirm that the abortion is complete (various options)*
    - *Refer for or provide diagnose and treat complications if any*
    - *Provide information/services on post abortion contraception*
    - *Post abortion care (PAC), protection from STI*
- ***Constellation of services***
  - - *PAC counselling and services*
    - *Screening for STIs including HIV, HPV and cervical cytology etc.*
    - *Referral for legal advice, family counselling*

**Individuals (users and non-users/partners/peer groups)** *(Pregnant individuals and their support networks are aware & informed about their rights, services, and methods available for abortion)*

- *Awareness of law/rights/methods/service locations*
  - - *Awareness of methods (MA/MVA/EVA)*
    - *Awareness of availability of services (nearest service delivery point / nearest source of medical abortion drug)*
    - *Awareness of trusted sources of information*
- *Equitable access to services*
  - - *By socio-demographic characteristics - Age, education, ethnicity/religion, attitudes towards abortion, income*
